# Identification of microRNA-like RNAs from *Trichoderma asperellum* DQ-1 during its interaction with tomato roots using bioinformatic analysis and high-throughput sequencing

**DOI:** 10.1371/journal.pone.0254808

**Published:** 2021-07-22

**Authors:** Weiwei Wang, Fengtao Zhang, Jia Cui, Di Chen, Zhen Liu, Jumei Hou, Rongyi Zhang, Tong Liu

**Affiliations:** 1 Key Laboratory of Green Prevention and Control of Tropical Plant Diseases and Pests (Hainan University), Ministry of Education, Haikou, Hainan, PR China; 2 Key Laboratory of Germplasm Resources of Tropical Special Ornamental Plants of Hainan Province, College of Forestry, Haikou, Hainan, PR China; Louisiana State University, UNITED STATES

## Abstract

MicroRNA-like small RNAs (milRNAs) and their regulatory roles in the interaction between plant and fungus have recently aroused keen interest of plant pathologists. *Trichoderma* spp., one of the widespread biocontrol fungi, can promote plant growth and induce plant disease resistance. To investigate milRNAs potentially involved in the interaction between *Trichoderma* and tomato roots, a small RNA (sRNA) library expressed during the interaction of *T*. *asperellum* DQ-1 and tomato roots was constructed and sequenced using the Illumina HiSeq^TM^ 2500 sequencing platform. From 13,464,142 sRNA reads, we identified 21 milRNA candidates that were similar to other known microRNAs in the miRBase database and 22 novel milRNA candidates that possessed a stable microRNA precursor hairpin structure. Among them, three milRNA candidates showed different expression level in the interaction according to the result of stem-loop RT-PCR indicating that these milRNAs may play a distinct regulatory role in the interaction between *Trichoderma* and tomato roots. The potential transboundary milRNAs from *T*. *asperellum* and their target genes in tomato were predicted by bioinformatics analysis. The results revealed that several interesting proteins involved in plant growth and development, disease resistance, seed maturation, and osmotic stress signal transduction might be regulated by the transboundary milRNAs. To our knowledge, this is the first report of milRNAs taking part in the process of interaction of *T*. *asperellum* and tomato roots and associated with plant promotion and disease resistance. The results might be useful to unravel the mechanism of interaction between *Trichoderma* and tomato.

## Introduction

MicroRNAs (miRNAs), small non-coding RNAs and approximately 22 nt in length, can negatively regulate gene expression and have been discovered in animals and plants [1]. In animals, miRNAs interact with their target genes through imprecise base pairing, leading to inhibition of translation [2]. However, in plants, miRNAs often repress target mRNAs by interacting with their target genes through near-perfect complementarity [3]. Recently, several research groups have confirmed the small RNAs (sRNAs) synthesis pathway in filamentous fungi such as *Neurospora crassa* [4], *Mucor circinelloides* [5], and *Magnaporthe oryzae* [6]. Two known milRNAs (microRNA-like small RNAs) and 42 novel milRNA candidates were identified in *Sclerotinia sclerotiorum* and found to have no homology to miRNAs from plants and animals, suggesting that milRNAs may have evolved independently in fungi [7]. MilRNAs have also been reported and validated in some species of *Trichoderma*. For example, RNAi-mediated gene silencing was performed in *T*. *reesei*, an industrial strain known for secreting cellulolytic enzymes, and the results revealed the presence of the RNA-induced silencing complex (RISC) machinery and milRNAs in this filamentous fungus [8]. Furthermore, thirteen milRNAs from *T*. *reesei* induced and uninduced by cellulose were examined by Solexa sequencing, and these milRNAs may play a role in the growth and cellulase production of *T*. *reesei* [9].

Recently, milRNAs were reported to possess the capacity of transboundary regulation during the interactions between plants and pathogenic fungi. For instance, a specific milRNA named *Pst-milR1* that simulated the function of a plant miRNA was identified in wheat stripe rust. *Pst-milR1* specifically targeted the *Pathogenesis Related Protein* 2 (*PRP*2) gene of wheat to suppress the host immune system and enhance the pathogenicity of stripe rust [10]. Likewise, fourteen sRNAs in *M*. *oryzae* were predicted to target rice genes associated with growth and disease defense [11]. In addition, many studies have suggested that plant miRNAs can be transferred to pathogenic fungi to regulate pathogen virulence. For example, *Arabidopsis thaliana* sRNAs can be transmitted to *Botrytis cinerea* through extracellular vesicles, weakening the pathogenicity of the pathogen [12]. Cotton plants produce microRNA 166 and microRNA 159 and export them to the hyphae of *Verticillium dahliae* to silence specific fungal virulence genes [13].

*Trichoderma* spp. as promising biocontrol agents can improve the plant rhizosphere environment, promote plant growth and development, and increase plant disease resistance [14]. However, the mechanisms of *Trichoderma* interacting with roots to promote tomato growth and induce tomato disease resistance have not been elucidated clearly [15–17]. *T*. *asperellum*, which is a widely used *Trichoderma* biocontrol agent, takes an important role in controlling *Fusarium* wilt disease and promoting growth in tomato [18–20]. Depending on the previous work, our group also found that *T*. *asperellum* could induce disease resistance and promote growth in tomato and many other crops (data not shown). To the best of our knowledge, no milRNAs have been reported in *T*. *asperellum*, particularly with respect to its interaction with tomato roots. Therefore, in this study, we use the deep sequencing technology and bioinformatic analysis to identify milRNA candidates from *T*. *asperellum* DQ-1 interacting with tomato roots. Potential transboundary milRNAs involved in this interaction were predicted. This project could provide information to clarify the role of milRNAs in promoting root growth and inducing disease resistance during the interaction between *T*. *asperellum* and tomato.

## Materials and methods

### *T*. *asperellum* strain, plant material and sample collection

*T*. *asperellum* strain DQ-1, which was isolated and identified by our lab, stored in the Engineering Center of Agricultural Microbial Preparation Research and Development of Hainan (Hainan University) and cultured on potato dextrose agar (PDA) medium. *T*. *asperellum* spore suspension (1×10^7^ cfu/mL) was prepared with sterile water. and added evenly onto the tomato roots.

Seeds of tomato (inbred line “Xinfan 2”) were kindly provided by Associate Professor Yonghua Liu (College of Horticulture, Hainan University). 150 seeds (50 seeds/replicate, three replicates) were surface sterilized with 0.1% sodium hypochlorite for 10 mins and rinsed for five times with sterile water under aseptic conditions, then placed on moistened filter paper cultured in the dark at 28°C for 48 h for germination. The germinated seeds were sown in growth medium consisting of vermiculite and perlite (3:1, v/v) and placed in a growth chamber at 28°C with a 12 h light-12 h dark photoperiod and relative humidity of 80%. After 4 weeks, uniform tomato seedlings (20 seedlings/replicate) were selected and *T*. *asperellum* spore suspension (1×10^7^ cfu/mL) prepared were added evenly onto the tomato roots. The roots were wrapped with tinfoil and incubated in the growth chamber (25°C, 80%RH). *T*. *asperellum* was collected from tomato roots at 0, 6, 12, 24, and 48 h after inoculation by washing the roots with sterile water. The rinse solutions were centrifuged at 12,000 rpm for 15 mins and the supernatants were discarded. The samples were quickly frozen with liquid nitrogen and stored in an ultra-low temperature freezer at −80°C.

### RNA extraction, sRNA library construction, and high-throughput sequencing

For the screening and identification of sRNAs from *T*. *asperellum* DQ-1 interacting with tomato roots, total RNAs were extracted from samples collected at different time points (0, 6, 12, 24, and 48 hours post-infection, hpi) using the Trizol reagent (Invitrogen, CA, USA) and according to the manufacturer’s instructions. The extracted RNAs from the five interaction periods were mixed together and used to construct a sRNA library for sequencing. The absorbance at 260 and 280 nm was measured using the NanoDrop ND-1000 spectrophotometer (NanoDrop Technologies, Wilmington, DE, USA) to photometrically assess RNA concentration. The sRNA fragments were enriched using the PEG8000 precipitation method [21], and sRNAs 18–30 nt in length were isolated using denatured 15% polyacrylamide gel electrophoresis. The purified sRNAs were ligated to Illumina’s proprietary 3′ and 5′ adapters and amplified by reverse transcription polymerase chain reaction (RT-PCR). Finally, the purified cDNA fragments were sequenced on the Illumina HiSeq^TM^ 2500 platform (Gene Denovo Biotechnology Co., Guangzhou, China).

### Data processing

Because raw Illumina fastq data always contain reads with adapter sequences or low-quality bases, the original data were filtered using TRIMMOMATIC v0.39 [22], FASTQC v0.118 [23] and the FASTX_TOOLKIT v0.013 [24] on the basis of the following criteria: (1) removal of reads containing more than one low-quality base (Q-value≤20) or unknown nucleotides (N), (2) removal of reads without 3′ adapters, (3) removal of reads that contained 5′adapters, (4) removal of reads with no sRNA fragment between 3′ and 5′ adapters, (5) removal of reads that contained polyA in the sRNA fragment, and (6) removal of reads shorter than 18 nt (adapters not included). The higher-quality unique reads were mapped to the *T*. *asperellum* CBS 433.97 genome (https://mycocosm.jgi.doe.gov/Trias1/Trias1.home.html) using BOWTIE2 v2.4.1 and SAMTOOLS v1.10 to obtain alignment position information, remove redundant sRNA sequences, and filter out degraded mRNA fragments [25, 26]. Unique sequences, including rRNA, small cytoplasmic RNA (scRNA), small nucleolar RNA (snoRNA), small nuclear RNA (snRNA), and tRNA, were annotated based on BLASTN v2.9.0 searches (E-value 0.01) of the GenBank database (release 209.0) and the Rfam database (11.0). In addition, REPEATMASKER v4.09 was used to filter out reads that derived from the repeat-associated region on the *T*. *asperellum* genome [27].

### Prediction and expression analysis of potential milRNAs

Due to lacking of data on miRNAs from *Trichoderma* spp. in the miRBase database (release 21), all the unique reads (excluding rRNA, scRNA, snoRNA, snRNA, tRNA, mRNA degradation fragments, and repeat sequences) were compared with miRNA sequences and precursor sequences from other species in the authoritative miRBase database to screen the known milRNA candidates using Bowtie2. For the prediction of novel milRNAs, MIREAP v0.20 was used in this study [28]. Based on the locations of unannotated sRNAs in the *T*. *asperellum* genome, all potential precursors with stable hairpin-like structures were identified using Mireap based on the following criteria: (1) minimum miRNA sequence length of 18 nt, (2) maximum miRNA sequence length of 25 nt, (3) minimum miRNA reference sequence length of 20 nt, (4) maximum miRNA reference sequence length of 23 nt, (5) maximum copy number of miRNA on reference of 20, (6) maximum free energy allowed for an miRNA precursor of 18 kcal/mol, (7) maximum space between miRNA and miRNA* (complementary sequence of miRNA in the precursor, the same below) of 300 nt, (8) minimum space between miRNA and miRNA* of 16 nt, (9) maximum bulge between miRNA and miRNA* of 4 nt, (10) maximum asymmetry of miRNA/miRNA* duplex of 4 nt, and (11) miRNA precursor flank sequence length of 20 nt.

The expression levels of known and novel milRNAs were calculated and normalized to transcripts per million (TPM) using RSEM v1.1.17 [29]. The value of TPM was calculated using the equation:

TPM = Actual miRNA counts/ (Total counts of clean reads×10^6^).

### Stem-loop RT-qPCR

Three milRNA candidates (milR001-5p, milR008-3p, and milR004-5p) were randomly selected from the identified milRNAs, and their expression levels at different time points during the *Trichoderma*–tomato root interaction (0, 6, 12, 24, and 48 hpi) were measured using stem-loop RT-qPCR. Total RNAs were extracted from each sample and treated with DNase I to remove DNA templates [30]. The mature milRNAs were reverse transcribed to complementary DNA (cDNA) with specific stem-loop RT primers designed in miRprimer v2.0 [31]. The reverse transcription reactions (10 μl) contained 1 μl RNA (200 ng), 2 μl 5× Primescript^®^ buffer (Clontech, USA), 1 μl stem-loop RT primer (10 μM), 0.5 μl Primescript^®^ RT Enzyme Mix I (5U) (Clontech, USA), and 5.5 μl RNase-free dH_2_O. The qRT-PCR reaction mixture (25 μl) included 1 μl RT product, 1×SYBR premix EX Taq Ⅱ^®^ (Takara, Dalian, China), 200mM forward primer, and 200mM reverse primer. The reaction conditions of qPCR were as follows: an pre-denaturation for 15 min at 95°C, followed by 40 cycles of denaturation at 94°C for 20 s, annealing and extension at 58°C for 34 s, and the progress of the reaction was monitored through the Eppendorf Mastercycler RealPlex^®^ Detection System. The milRNA expression levels were calculated using the comparative Ct method (ΔΔCt). The α-tubulin gene was used as the internal reference and the expression level of each milRNA at 0 hpi was used as the normalization control. The experiment was repeated three times. The RT primers and qPCR primers were presented in S1 Table.

### Target prediction and functional enrichment analysis of *Trichoderma* milRNAs

The target genes of *Trichoderma* milRNAs identified in *T*. *asperellum* CBS 433.97 genome (https://mycocosm.jgi.doe.gov/Trias1/Trias1.home.html) were predicted using PATMATCH v1.2 [32], and verified according to the following rules: (1) no more than four mismatches in the complementary relationship between the miRNA and its target (G-U bases counted as 0.5 mismatches), (2) no more than two adjacent mismatches in the miRNA/target duplex, (3) no adjacent mismatches in positions 2–12 of 5′ miRNA in the miRNA/target duplex, (4) no mismatches in positions 10–11 of the miRNA/target duplex, (5) no more than 2.5 mismatches in positions 1–12 of 5′ miRNA in the miRNA/target duplex, and (6) the minimum free energy (MFE) of the miRNA/target duplex ≥60% of the MFE of the miRNA bound to its perfect complementary sequence. The target genes of these milRNAs were also collected in *Trichoderma* spp. and GO term and KEGG pathway annotation were performed using the DAVID gene annotation tool (http://david.abcc.ncifcrf.gov/UTH) to investigate the functions of the milRNA target genes [33].

### Prediction and functional analysis of potential transboundary milRNAs

To identify the potential transboundary miRNAs from *T*. *asperellum*, all the milRNAs screened above were mapped to the tomato genome from the NCBI Refseq database (accession GCF_000188115.4_SL3.0) using blastall. For milRNAs that mapped perfectly to the tomato genome, 100 bp sequences up- and downstream of the matched region in the tomato genome were submitted to the MFOLD web server (http://unafold.rna.albany.edu/?q=mfold/RNA-Folding-Form) with a minimal free energy (MFE) value of −85 kcal mol^−1^ to check whether the milRNA precursor sequences could form stable secondary structures in the tomato genome [34]. The milRNA precursor sequences that did not form stable secondary structures in the tomato genome and did not perfectly match the tomato genome were used to predict tomato target genes according to the following steps.

The prediction of potential transboundary milRNAs was performed using PSRNATARGET (http://plantgrn.noble.org/psRNATarget/) with the NCBI tomato (*Solanum lycopersicum*) transcript library (accession GCF_000188115.4_SL3.0) to identify milRNAs that targeted tomato [35]. To verify the prediction results, PSROBOT and TARGETFINDER (https://github.com/carringtonlab/TargetFinder) were also used to examine whether the screened milRNAs targeted the tomato transcript library [36, 37].

More stringent parameters for the PSRNATARGET program were used to rapidly identify milRNAs that may have been involved in transboundary gene regulation. The specific parameters were: (1) no gaps or bulges were permitted within the sRNA–target alignment, (2) the tenth nucleotide of the sRNA was required to perfectly match its target, (3) one mismatch or two wobbles at most were permitted from the seed region on the mature sRNA sequence from the second to the twelfth nucleotide, (4) a maximum of two continuous mismatches was permitted, and (5) the threshold cut-off value was set to 4.0.

In PSROBOT, we performed milRNA target prediction using the following criteria: (1) the target penalty score threshold was set to 4.0, (2) the second to seventeenth nucleotides from the 5′ essential sequence served as the seed region, (3) no gaps or bulges were permitted within the sRNA–target alignment, and (4) at most one gap or bulge was permitted after the seventeenth nucleotide of the complementary sequence. In TargetFinder, the miRNA target gene matching score threshold was set at 4.0, and the other parameters were set according to the author’s recommendation.

If all three programs predicted that a milRNA from *Trichoderma* had a target gene in tomato, then it was considered to be a potential transboundary milRNA. To minimize false positives, we only selected target genes predicted by all three programs, and we annotated the functions of the milRNA target genes using Blast2GO (https://www.blast2go.com/) [38].

## Results

### Identification of sRNAs in *T*. *asperellum*

**Fig 1:**
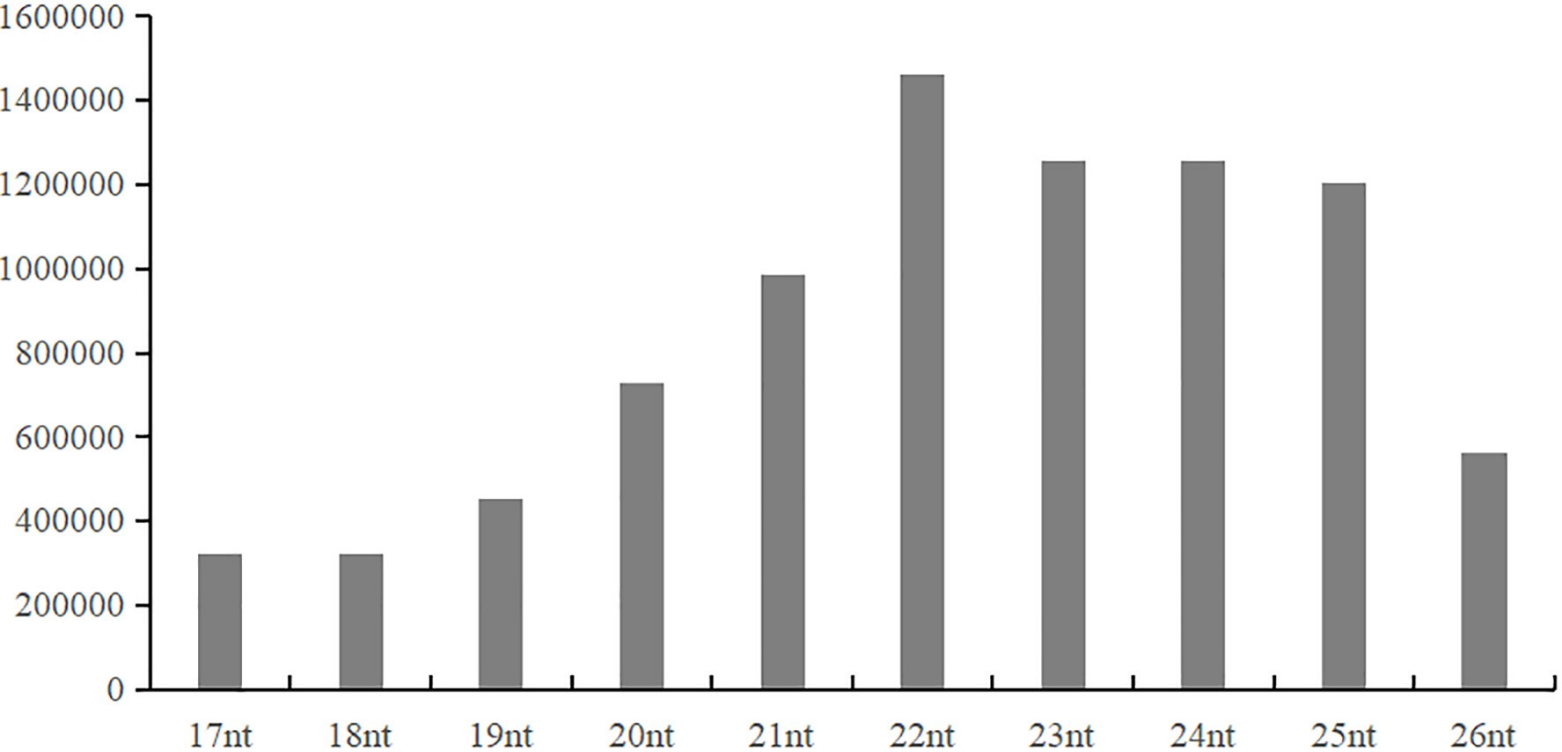
Length and frequency distribution of sRNAs from a cDNA library of *T*. *asperellum* DQ-1 interacting with tomato roots.

To investigate potential miRNAs from *T*. *asperellum*, sRNAs produced during different stages when interacted with tomato roots (0, 6, 12, 24, and 48 hpi) were extracted, and cDNA libraries of sRNAs (18–30 nt) were constructed and sequenced on the Illumina HiSeq platform (Gene Denovo Biotechnology Co, Guangzhou, China). A total of 15,049,834 clean sRNA reads were produced. Among them, 14,851,368 high quality reads (98.68% of the clean reads) were obtained after the removal of 3′ adapter nulls (0.19% or 27,899 reads), 5′ adapter contaminants (0.06% or 9,355 reads), insert nulls (0.66% or 98,745 reads), and polyA sequences (0.001% or 193 reads). In total, 13,464,142 clean tags representing 1,858,454 unique sRNA sequences were obtained after removal reads smaller than 18 nt from the high quality reads (Table 1). The majority of unique sRNA sequences ranged from 17 to 26 nt in length, and sequences of 22 nt were the most abundant, similar to the distribution pattern of sRNAs in plants and animals (Fig 1) [39, 40].

**Table 1:** Mapping statistical analysis of sRNAs sequences in *T*. *asperellum* DQ-1.

Category	Reads count	Percent (%)
clean_reads	15049834	100%
high_quality	14851368	98.68%
3’adapter_null	27899	0.19%
insert_null	98745	0.66%
5’adapter_contaminants	9355	0.06%
smaller_than_18nt	1251034	8.42%
polyA	193	0.00%
clean_tags	13464142	90.66%

### Annotation and classification of sRNAs

To identify and classify sRNAs, all unique reads that mapped to the *T*. *asperellum* genome were characterized based on their Blastall hits in the GenBank and Rfam databases. The results showed that 107,491 unique sRNAs were abundant non-coding RNAs (including rRNA, tRNA, snRNA, and snoRNA), 56,007 unique sRNAs were derived from the mRNAs/exons/introns of *T*. *asperellum*, and 31 unique reads (representing 32 sequence reads) were derived from repetitive regions (Table 2). A large number of unannotated sRNA sequences (1,694,925 unique reads representing 11,725,692 sequence reads) implied that *T*. *asperellum* has a high degree of sRNA sequence complexity and provided more resources for identifying potential milRNAs.

**Table 2:** The summary distribution of total and unique sRNA sequences among different categories in *T*. *asperellum* DQ-1.

sRNA category	Total sRNA	Percent (%)	Unique sRNA	Percent (%)
Clean reads	13,464,142	100	1,858,454	100
Exon antisense reads	6,565	0.05	4,021	0.22
Exon sense reads	65,299	0.48	50,618	2.72
Intron antisense reads	232	0.001	200	0.01
Intron sense reads	2,620	0.02	1,168	0.06
tRNA reads	340,736	2.53	19,200	1.03
rRNA reads	1,312,667	9.75	86,509	4.65
snRNA reads	8,390	0.06	1,329	0.07
snoRNA reads	1,909	0.01	453	0.02
repeat-associated reads	32	0.00	31	0.00
unannotated reads	11,725,692	87.06	1,694,925	91.2

### Identification of potential milRNAs from *T*. *asperellum*

**Fig 2:**
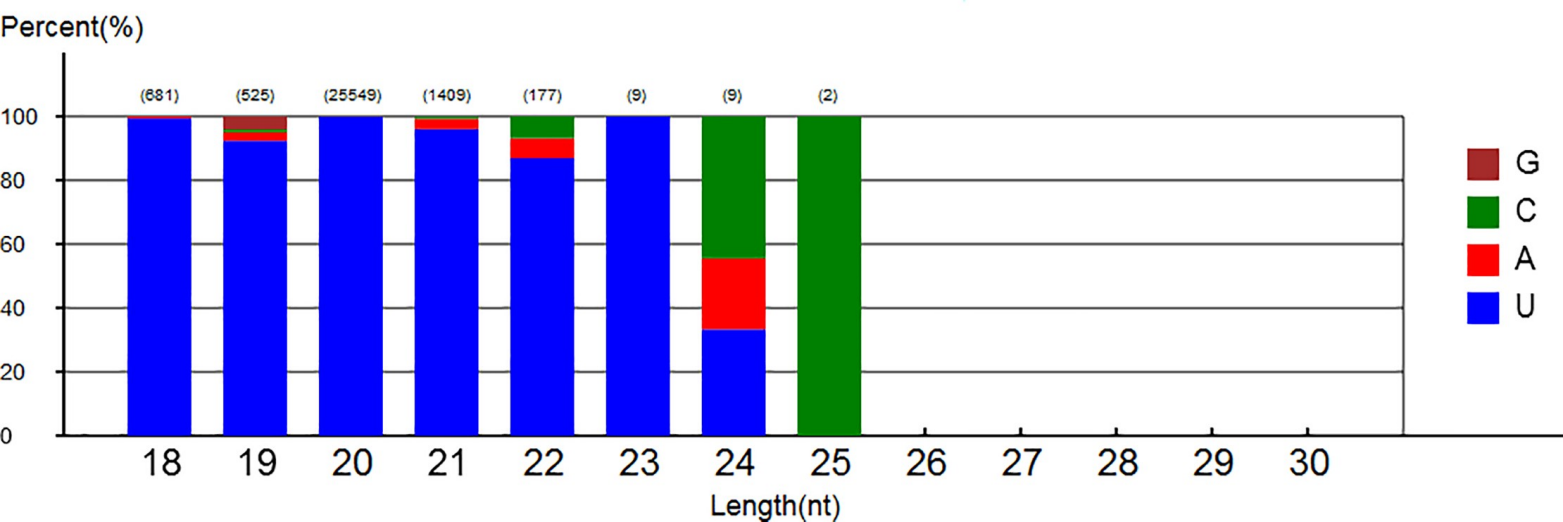
The first nucleotide bias of predicted novel milRNAs from *T*. *asperellum* DQ-1 sRNA library interacting with tomato roots.

**Fig 3:**
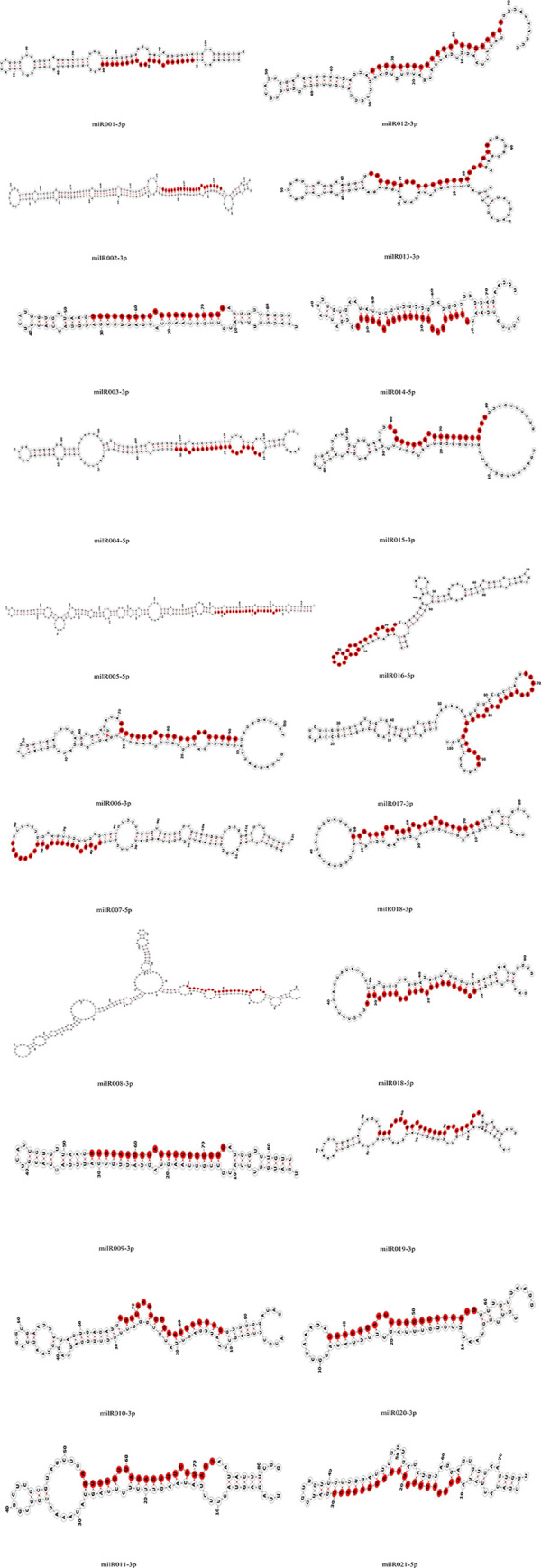
Hairpin structures of 22 novel milRNA precursor sequences from *T*. *asperellum* DQ-1 predicted using Mireap software. Mature milRNAs in precursor sequences are marked in red.

Based on comparisons with miRNAs from other species in miRBase using bowtie2 software, 21 unique reads representing 1,801 clean reads were identified as known milRNA candidates. Twenty-two novel milRNA candidates were identified from 21 potential precursor milRNAs with stable hairpin structures and flanking nucleotide sequences in the genome using Mireap software (Table 3). Among the novel milRNAs, milR018-3p and milR018-5p originated from the same precursor. The first base of these novel milRNAs had a strong bias toward U (Fig 2), consistent with miRNAs from other fungi such as *Metarhizium anisopliae* [41] and *Aspergillus flavus* [42]. The precursor sequences of the 22 novel milRNAs were submitted to the RNAfold website (http://rna.tbi.univie.ac.at//cgi-bin/RNAWebSuite/RNAfold.cgi) [43] to predict their stem-loop structures, and were then edited using the website’s forma structure visualization tools (Fig 3).

**Table 3:** Novel milRNAs from *T*. *asperellum* DQ-1 identified by high-throughput sequencing and characteristics of pre-milRNA sequence.

Novel milRNAs	Sequences(5’-3’)	Length(nt)	Total reads	Location of precursor	Length of precursors (nt)	MFE(kcal mol-1)
milR001-5p	TGAAACCGCGAACAAACTTG	20	24796	scaffold_3:1682263:1682370:+	108	-49.4
milR002-3p	TCCACCGTCGCCAGCTGCAG	20	2366	scaffold_2:1320903:1321074:+	172	-110.1
milR003-3p	TCGTAATACAGCTTGTCGGG	20	398	scaffold_2:1321094:1321176:+	83	-48.3
milR004-5p	TAGGAAGAGAGCCATTTACGC	21	385	scaffold_2:1267024:1267161:-	138	-61.3
milR005-5p	TGCTGACGGAGCTAAGGTACGC	22	373	scaffold_2:1256427:1256640:-	214	-89.2
milR006-3p	AGGGATAGGGAGAGGAAGGGA	21	22	scaffold_3:2254108:2254208:+	101	-24.8
milR007-5p	TGTGAGAACCTGGGCATGACT	21	10	scaffold_8:97829:97948:-	120	-40.9
milR008-3p	TCCCCGTTGCGATGGTACTCCG	22	8	scaffold_10:1024744:1024984:-	241	-59.1
milR009-3p	TCGTAATACAGCTTGTCGGG	20	398	scaffold_2:1321094:1321176:+	83	-48.3
milR010-3p	AGCCAAATGTGGAGCTCGAT	20	7	scaffold_10:304729:304824:+	96	-30.6
milR011-3p	AGTTGGTTAGAGCTTCGTACT	21	177	scaffold_10:446984:447066:-	83	-20.4
milR012-3p	CTTGGTGTTGGTGCAGTAGACA	22	15	scaffold_11:1337159:1337256:+	98	-29.4
milR013-3p	AGGACTGGTGCCTGGCGCCTCG	22	5	scaffold_11:554520:554615:-	96	-27.1
milR014-5p	CGGCAGATTAGTAGTCAGGTGG	22	5	scaffold_12:175206:175281:-	76	-18.2
milR015-3p	ACGGCAAGTGCACTGACATC	20	10	scaffold_18:34970:35058:+	89	-19.6
milR016-5p	CGACGTCGTGTGGATGGAGCCT	22	14	scaffold_1:1455488:1455586:+	99	-21.4
milR017-3p	ACGAGATGGTTGGTGACAAGGACA	24	11	scaffold_4:1392134:1392234:-	101	-22.7
milR018-3p	CGTGGGCGGGATGGCTCGGCATA	23	6	scaffold_8:1880833:1880914:+	82	-23.9
milR018-5p	TTGCCGGCTCTCCAACTCTCGA	22	7	scaffold_8:1880833:1880914:+	82	-23.9
milR019-3p	CGGAACCGTCGGAAGAAACATTCA	24	35	scaffold_8:1949994:1950081:+	88	-23.8
milR020-3p	TGTGAGAACCTGGGCATGACT	21	10	scaffold_8:97908:97975:-	68	-29.7
milR021-5p	TCGGATGTGTTGAGTTGAGC	20	9	scaffold_9:1217272:1217346:-	75	-24.2

### milRNA expression profiles and RT-qPCR verification

The expression levels of all known and novel milRNAs were measured and normalized to transcripts per million (TPM) using RSEM software based on their read counts in the library. The TPM values of novel milRNAs varied approximately between 144 and 713552, with milR001-5p showing the highest TPM value (S3 Table). Likewise, the TPM values of the known milRNAs varied approximately between 144 and 31194, with miR166-y showing the highest TPM value (S2 Table). These results suggested that the expression levels of different milRNAs were very different during the interaction of *Trichoderma* and tomato roots.

**Fig 4:**
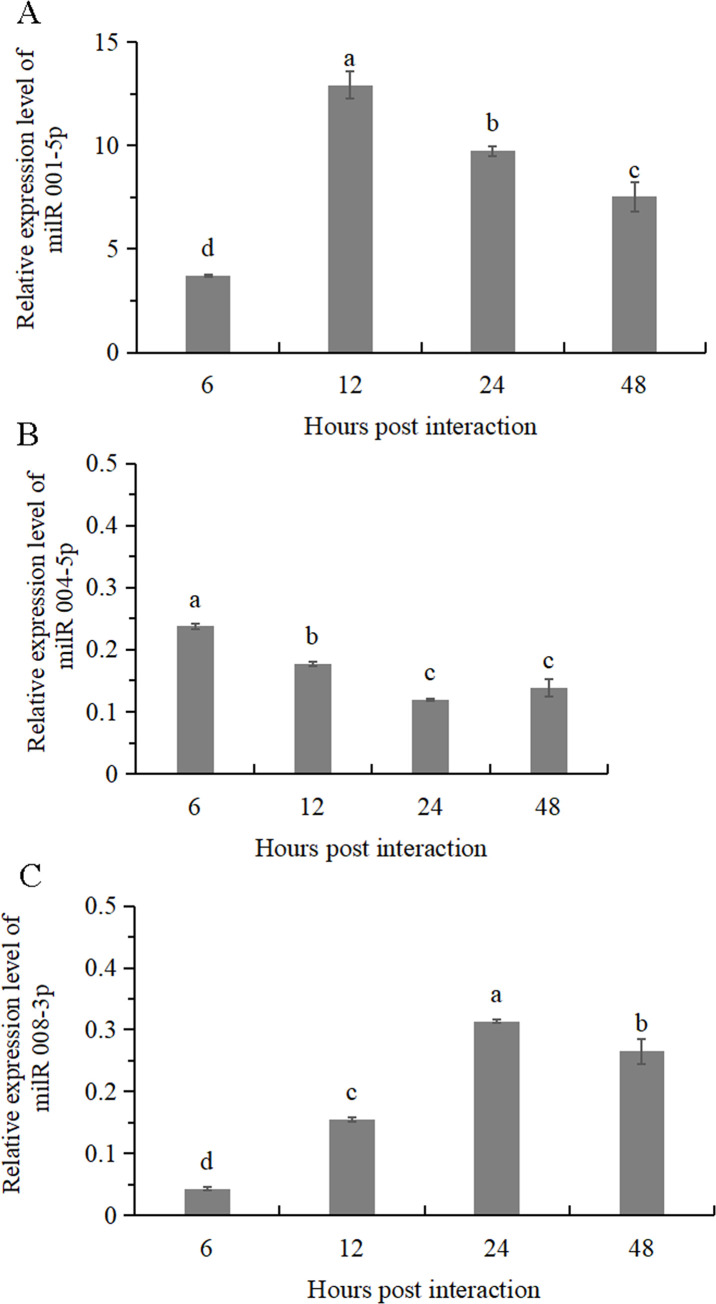
Expression level of three milRNAs candidates of *T*. *asperellum* DQ-1 at different time points (6, 12, 24 and 48 hpi) during the interaction with tomato roots using stem-loop RT-PCR. A-C were for the three random milRNAs candidates milR001-5p, milR004-5p and milR008-3p, respectively. Expression levels were calculated by the comparative Ct method (ΔΔCt), the α-tubulin as the internal reference gene, and the expression level of each milRNA at 0 hpi was used as a normalization control.

To investigate the expressions of the milRNA at different time points in the interaction, three milRNA candidates (milR001-5p, milR004-5p, and milR008-3p) were selected randomly and their expression profiles were analyzed through stem-loop RT-qPCR (Fig 4). The expression levels of milR001-5p, milR004-5p, and milR008-3p were highest at 12, 6, and 24 hpi, respectively, and declined thereafter. These results implied that milR001-5p may have an important role in the early stage of interaction between *Trichoderma* and tomato roots. In addition, the expression level of milR001-5p was higher than milR004-5p and milR008-3p, suggesting that they may have a distinct regulatory role in the interaction between *Trichoderma* and tomato roots.

### Prediction and functional enrichment analysis of *T*. *asperellum* milRNA target genes in *T*. *asperellum*

**Fig 5:**
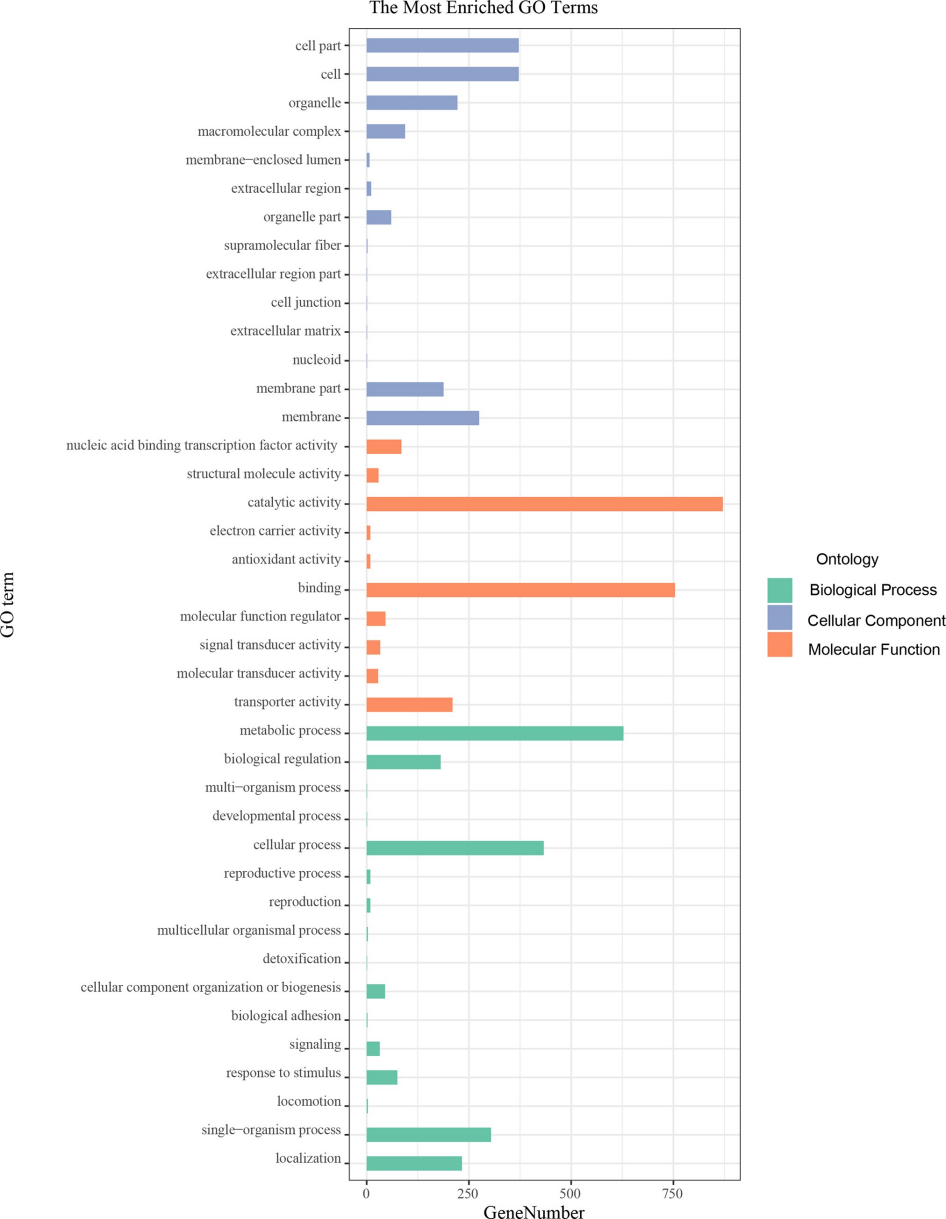
GO annotation of potential milRNA target genes in *T*. *asperellum* DQ-1. The results were grouped into three main categories: cellular component, molecular function, and biological process.

**Fig 6:**
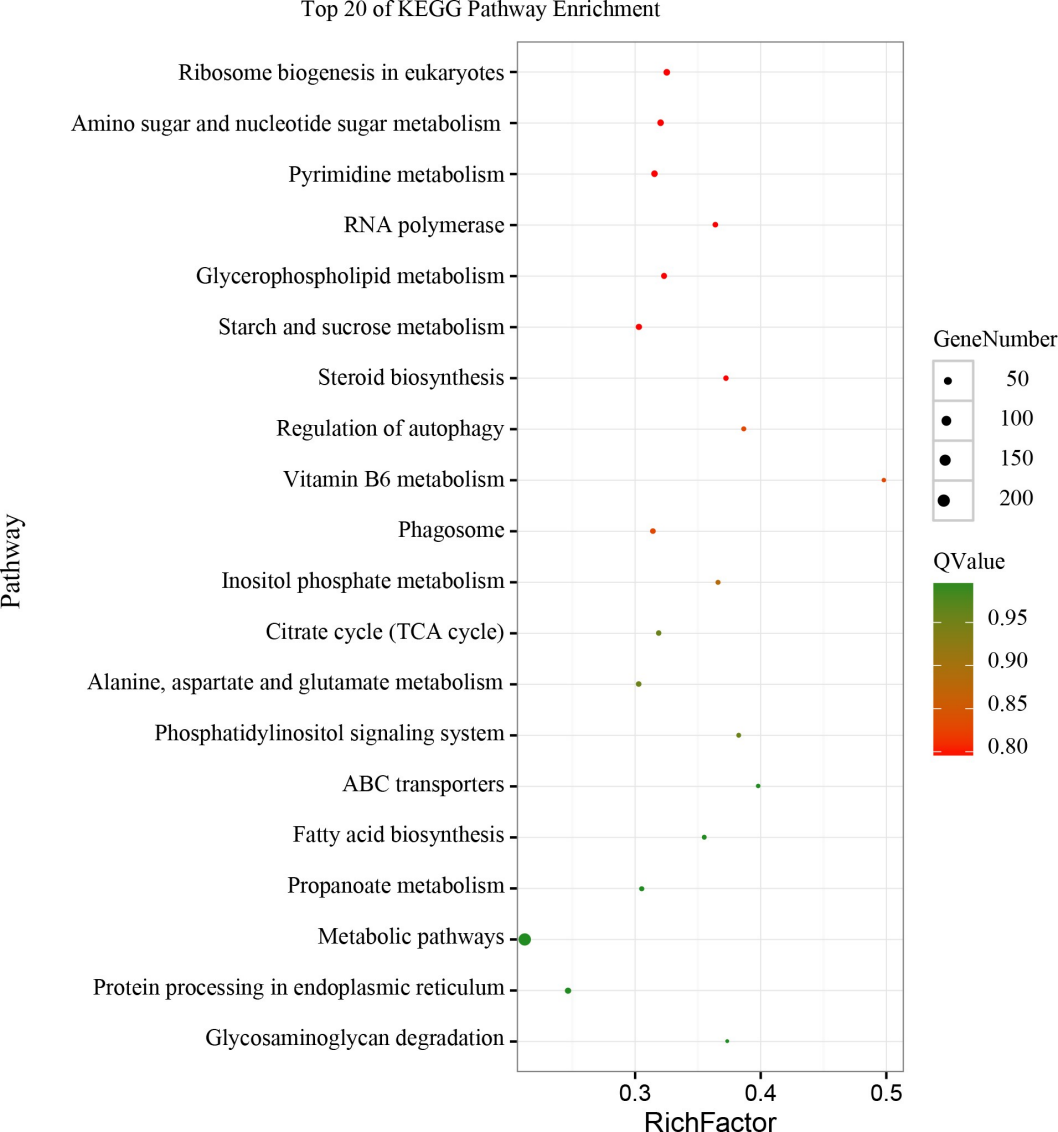
KEGG pathway enrichment of target genes of potential milRNA targets in *T*. *asperellum* DQ-1.

To better understand the regulatory roles of milRNAs during different stages of the interaction between *T*. *asperellum* and tomato, the potential target genes of all milRNAs were predicted using patmatch software. In total, 502 target genes for 16 known milRNAs and 30 target genes for 8 novel milRNAs were identified in the *T*. *asperellum* genome (S4 Table). The enrichment analysis of GO functions and KEGG pathways of target genes was performed using the DAVID gene annotation tool to (S5 and S6 Tables). The results suggested that the target genes of milRNAs were enriched for GO terms associated with the establishment of localization, anion transport, integral component of membrane, and ATPase activity (Fig 5), and the top 20 KEGG pathways of the milRNA target genes were mainly related to ribosome biogenesis, nucleotide sugar metabolism, and pyrimidine metabolism (Fig 6).

### Prediction and functional analysis of potential transboundary milRNAs

**Fig 7:**
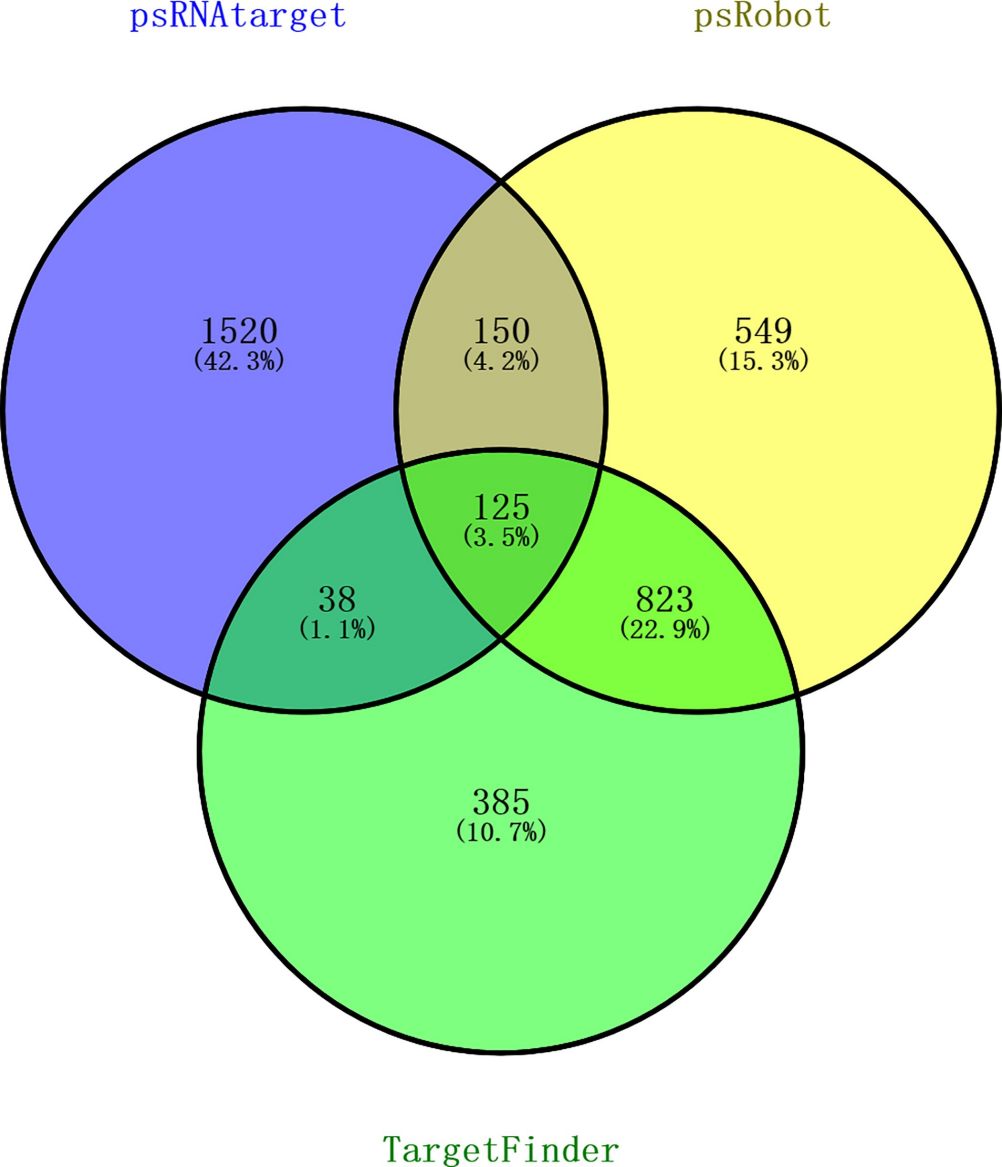
Venn chart of potential transboundary milRNAs target genes in tomato predicted using psRNAtarget, psRobot, and TargetFinder.

**Fig 8:**
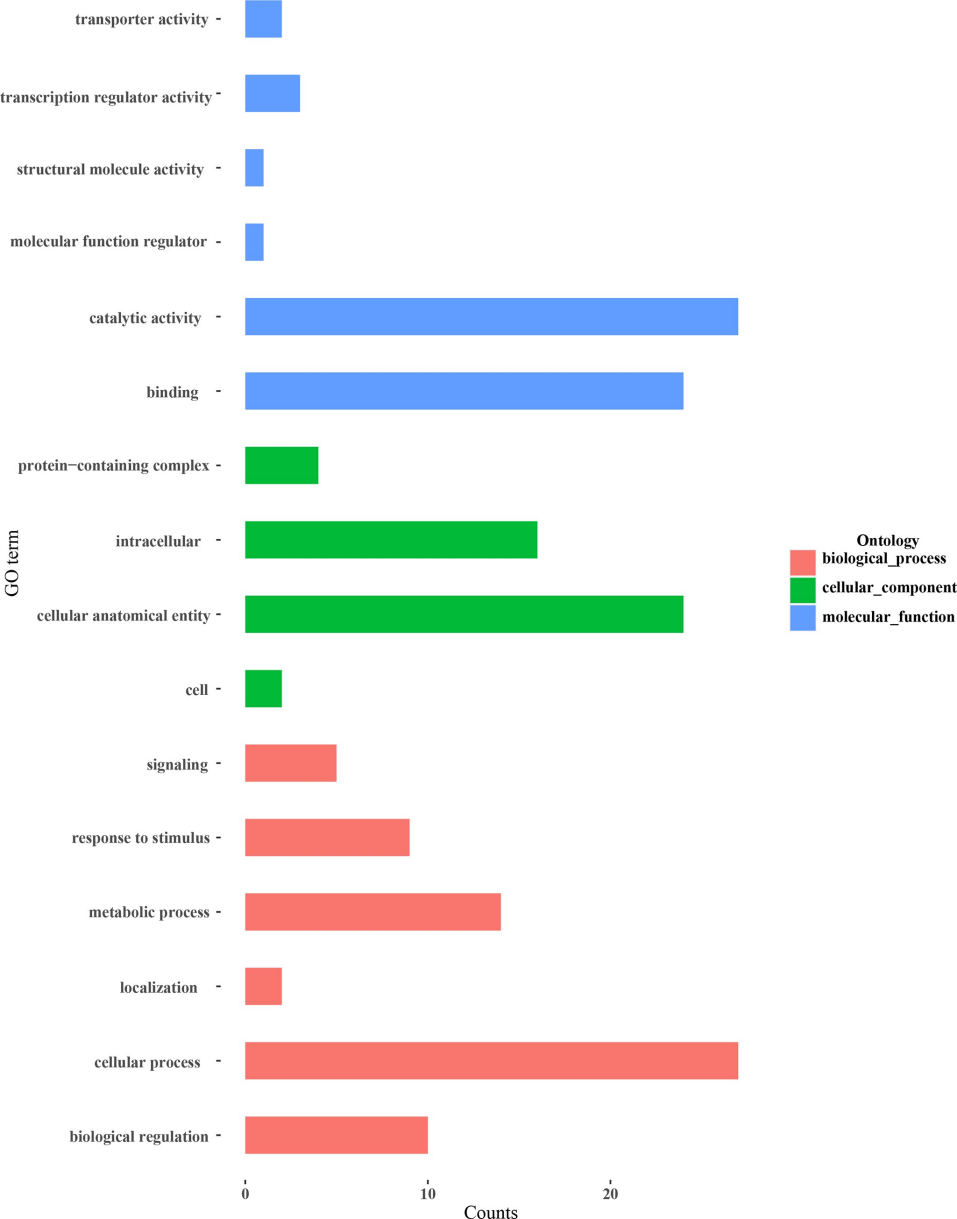
GO classification of potential transboundary milRNAs target genes in tomato.

To identify potential transboundary milRNAs expressed during the interaction between *T*. *asperellum* and tomato roots, all milRNAs that did not exist or form stable precursor sequences in tomato and could target tomato genes were predicted (S1 Fig). We found that 31 milRNAs were potentially capable of transboundary regulating tomato mRNAs during the interaction of *Trichoderma* and tomato roots. Specifically, 2,086, 1,765, and 1,463 target genes of these potential transboundary milRNAs were predicted using psRNAtarget (S7 Table), psRobot (S8 Table), and TargetFinder (S9 Table), respectively. Among these target genes, 125 common target genes associated with plant growth and development, disease resistance, seed maturation, and osmotic stress signal transduction were identified by all three software programs (Fig 7). These results provided milRNA resources for elucidating the mechanism of *Trichoderma* promoting root growth and inducing plant disease resistance during its interaction with tomato. We also analyzed the GO annotations of these target genes using Blast2GO (Fig 8) and found that the potential transboundary milRNAs may play a role in regulating biological processes such as catalytic activity, cellular process, cellular anatomical entity, and binding during the interaction of *Trichoderma* and tomato.

## Discussion

Fungal species of the genus *Trichoderma* are commonly used as promising biocontrol agents against phytopathogenic fungi, and some isolates of *Trichoderma* spp. can colonize successfully in tomato roots, showing growth promotion and disease resistance [44, 45]. Studies showed that *T*. *asperellum* strains TS-12 and TS-39 induce systemic resistance (ISR) against the tomato wilt pathogen *F*. *oxysporum* f. sp. *lycopersici* in association with the modulation of defense-related genes, chitinase (*SlChi3*), β-1,3-glucanase (*SlGluA*) and PR-1 (*SlPR-1*α) [19]. *T*. *asperellum* was used to combat *Ralstonia solanacearum* attack in tomato plants due to the activity of peroxidase (POX), phenylalanine ammonium lyase (PAL), polyphenol oxidase (PPO) and β-1,3-glucanase being induced significantly [46]. Tomatoes pre-treated with *T*. *harzianum* strains T908 and T908-5 reprogram the expression of genes associated with the salicylic acid signaling pathway and ethylene biosynthesis and exhibit decreased susceptibility to *Meloidogyne incognita* [47]. However, little is known about the function of milRNAs during the interaction between *Trichoderma* and tomato roots. In this article, to investigate the potential *Trichoderma* milRNAs playing roles in improving tomato growth and inducing disease resistance, total RNAs expressed at different stages in the interaction of *T*. *asperellum* and tomato roots were extracted and a sRNA library was constructed.

MicroRNAs (miRNAs) ranging from 18 to 30 nt in size and originating from a precursor with a structure of hairpin or stem-loop have been found to play an important role in the biological processes of animals and plants. Recently, milRNAs, which are similar to microRNAs, have been identified by deep sequencing, bioinformatics analysis, and northern blotting in filamentous fungi, such as *N*. *crassa* [4] and *A*. *fumigatus* [48]. In this study, we identified 21 known and 22 novel milRNAs from *T*. *asperellum* DQ-1 produced during its interaction with tomato roots using Illumina sequencing and bioinformatic analysis. We found that the sRNAs were 17–26 nt in length, with 22 nt as the most abundant, which was similar to the result reported in *T*. *reesei* [9]. In addition, the expression levels of milRNAs were varied during the process of interaction with tomato roots according to the result of qRT-PCR of three randomly selected milRNAs (milR001-5p, milR008-3p, and milR004-5p), which suggesting that milRNAs may play a distinct regulatory role in the interaction. This result was consistent with the reported findings that *Pst-milR1* as a novel milRNA in *Puccinia striiformis* (*Pst*) showed different expression pattern in different time in wheat leaves (Su11) inoculated with *Pst* race CYR31 (virulent) [10].

In recent years, some transboundary milRNAs between plants and fungi have been identified. Three sRNAs from *B*. *cinerea* (Bc-siR3.1, Bc-siR3.2, and Bc-siR5) were shown to silence genes involved in plant immunity through hijacking the host RNA interference (RNAi) machinery in *A*. *thaliana* and tomato [49]. Two microRNA-like sRNA candidates (pt-mil-RNA1 and pt-mil-RNA2) were discovered from *Puccinia triticina* during its interaction with wheat through deep sequencing and were predicted to target wheat genes that regulate specific biological processes, including defense-related pathways [50]. In this work, 31 potential transboundary milRNAs were predicted through bioinformatic analysis. And also, the target genes of the potential transboundary milRNAs were predicted in tomato using psRNAtarget, TargetFinder, and psRobot, some of which were involved in plant disease resistance, such as the coding sequences of bacterial spot disease resistance protein 4 (Bs4) [51, 52] and the transcription factor bHLH87 [53, 54], suggesting that these milRNAs may participate in the regulation of tomato disease resistance.

We found that one of the predicted target genes of novel milRNA m008-5p was an ethylene-responsive transcription factor ERF020-like transcript related to tomato defense against *B*. *cinerea* and induced by *Trichoderma* [55, 56]. The novel milRNA milR0001-3p was predicted to target the suppressor of *ABI3-5*, leading to plant growth promotion and seed maturation [57–59]. Additionally, the novel milRNA milR004-3p may target the coding gene of a MAPKKK-like serine/threonine kinase domain-containing protein that regulates signal transduction in response to osmotic stress [60]. Therefore, our findings suggested that these potential transboundary milRNAs might play a role in promoting plant growth and development, accelerating seed maturation, and regulating osmotic stress signal transduction during the interaction between *T*. *asperellum* DQ-1 and tomato, and milRNAs might be useful to unravel the mechanism of interaction between *Trichoderma* and tomato.

## Conclusion

In this study, a sRNA library expressed during the interaction of *T*. *asperellum* DQ-1 and tomato roots was constructed, and 21 milRNA candidates that were similar to other known microRNAs in the miRBase database and 22 novel milRNA candidates that possessed a stable microRNA precursor hairpin structure were identified. In total, 502 target genes for 16 known milRNAs and 30 target genes for 8 novel milRNAs were identified in the *T*. *asperellum* genome using patmatch software. The target genes of milRNAs were enriched for GO terms associated with the establishment of localization, anion transport, integral component of membrane, and ATPase activity, and the top 20 KEGG pathways of the milRNA target genes were mainly related to ribosome biogenesis, nucleotide sugar metabolism, and pyrimidine metabolism. The potential transboundary milRNAs from *T*. *asperellum* and their target genes in tomato were predicted by bioinformatics analysis, which revealed that several interesting proteins involved in plant growth and development, disease resistance, seed maturation, and osmotic stress signal transduction might be regulated by the transboundary milRNAs.

## Supporting information

S1 Fig Flowchart of potential transboundary milRNA prediction. Note: The numbers in parentheses represent the number of milRNA mature sequences.
(TIF)

S1 Table The primers for stem-loop RT-qPCR used in this study. (XLSX)

S2 Table Expression level of known milRNAs detected from *T*. *asperellum* DQ-1. (XLSX)

S3 Table Expression level of novel milRNAs detected from *T*. *asperellum* DQ-1. (XLSX)

S4 Table Target genes of all milRNAs were predicted by patmatch in *T*. *asperellum*. (XLSX)

S5 Table Go enrichment analysis of all milRNAs targets in *T*. *asperellum*. (XLSX)

S6 Table KEGG pathway enrichment of all milRNAs targets in *T*. *asperellum*. (XLSX)

S7 Table Prediction of potential transboundary milRNAs targets in tomato by psRNAtarget. (XLSX)

S8 Table Prediction of potential transboundary milRNAs targets in tomato by psRobot. (XLSX)

S9 Table Prediction of potential transboundary milRNAs targets in tomato by TargetFinder. (XLSX)
