# Supplementary figures and images for: Identification of microRNA-like RNAs from Trichoderma asperellum DQ-1 during its interaction with tomato roots using bioinformatic analysis and high-throughput sequencing
Source: PLoS One. 2021 Jul 22;16(7):e0254808. doi: 10.1371/journal.pone.0254808 (PMC8297844; doi:10.1371/journal.pone.0254808)

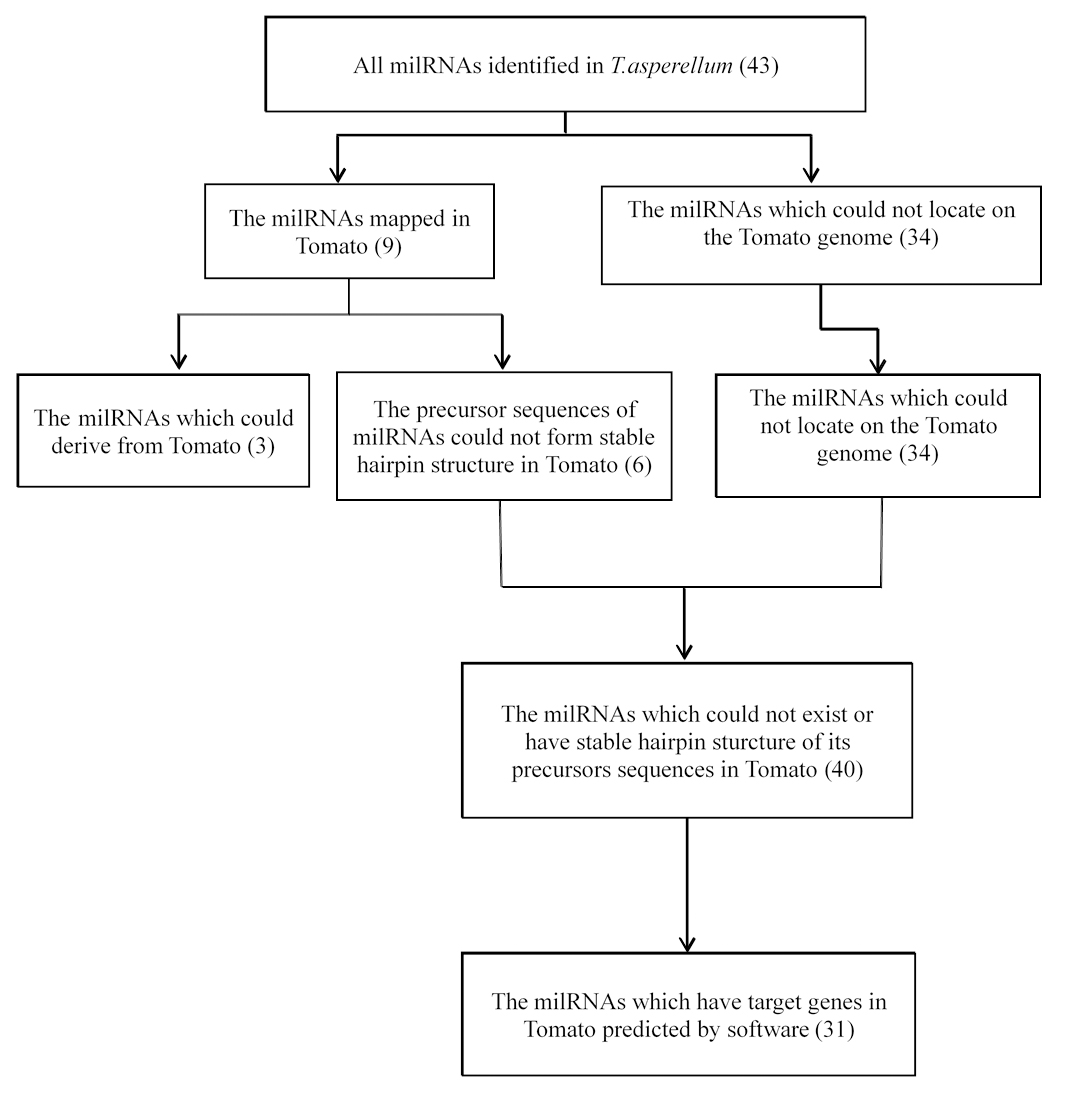

Supplement: S1 Fig — Note: The numbers in parentheses represent the number of milRNA mature sequences. (TIF) [file pone.0254808.s001.tif]
